# Intraovarian insertion of autologous platelet growth factors as cell-free concentrate: Fertility recovery and first unassisted conception with term delivery at age over 40

**DOI:** 10.18502/ijrm.v18i12.8030

**Published:** 2020-12-21

**Authors:** E. Scott Sills, Natalie S. Rickers, Samuel H. Wood

**Affiliations:** ^1^Reproductive Biology Group IVF, FertiGen CAG; San Clemente, California USA.; ^2^Department of Obstetrics and Gynecology, Palomar Medical Center; Escondido, California USA.; ^3^Gen 5 Fertility Center; San Diego, California USA.

**Keywords:** Ovarian rejuvenation, Platelet-rich plasma, Cytokines, Infertility, IVF.

## Abstract

**Background:**

The use of autologous platelet-rich plasma as an ovarian treatment has not been standardized and remains controversial.

**Case Presentation:**

A 41½-year old woman with diminished ovarian reserve (serum anti- Müllerian hormone = 0.163 mg/mL) and a history of 10 unsuccessful in vitro fertilization cycles presented for reproductive endocrinology consult. She and her partner declined donor oocyte in vitro fertilization. They were both in good general health and laboratory tests were unremarkable, except for mild thrombocytosis (platelets = 386K; normal range 150-379K) discovered in the female. The patient underwent intraovarian injection of fresh platelet-derived growth factor concentrate administered as an enriched cell-free substrate. Serum anti- Müllerian hormone increased by 115% within 6 wks of treatment. Spontaneous ovulation occurred the month after injection and subsequently the serum human chorionic gonadotropin was noted at 804 mIU/mL. Following an uneventful obstetrical course, a male infant was delivered at term without complication.

**Conclusion:**

This is the first description of intraovarian injection of enriched platelet-derived growth factors followed by unassisted pregnancy and live birth. As a refinement of conventional ovarian platelet-rich plasma therapy, this procedure may be particularly valuable for refractory cases where prognosis for pregnancy appears especially bleak. A putative role for thrombocytosis is also viewed in parallel with mechanisms of action as advanced earlier. With continued experience in ovarian application of autologous platelet growth factors, additional research will evaluate laboratory protocol/sample preparation, injection technique, and patient selection.

## 1. Introduction

It is well-known that as women age, both the quality and the quantity of eggs decline; the low ovarian reserve observed among older infertile patients occurs as an expected physiological consequence of normal ovarian senescence. In such cases, even the use of high-dose gonadotropin protocols is generally futile, leaving oocyte donation/IVF as the only clinically effective treatment (1-3).

As an investigational alternative to egg donation, the surgical placement of autologous platelet-rich plasma (PRP) into ovarian tissue first began to attract attention in 2016 (4). This pioneering technique of ovarian “rejuvenation” was followed by two publications describing similar use of PRP for poor-prognosis patients as a precursor to IVF. Specifically, four patients with undetectable or very low ovarian reserve (mean age 42 years) who had planned for donor egg treatment instead underwent the PRP treatment; all four patients developed blastocysts from their oocytes (5), one of them has since undergone thaw, transfer, and had a healthy term delivery. Experts in Greece also described three poor-responder IVF patients (mean age 38 years) with similar “revolutionary” responses (6). At least one patient who produced only embryos with genetic errors was able to achieve “ploidy rescue” following intraovarian injection of platelet-derived growth factors (PDGFs) before IVF, culminating in successful term live birth (7). Ovarian PRP has been formally evaluated in a descriptive pilot study including > 150 patients, where no significant change was observed in the serum AMH of most patients (8). However, the measured response rate after ovarian PRP (28%) approximates the overall IVF pregnancy rate in the USA. While ovarian treatment with growth factors has generally been framed as a precursor to IVF (5), almost no data exist on the reproductive outcome in the absence of IVF. Here, intraovarian injection of enriched, cell-free platelet growth factors followed by healthy term delivery - with no gonadotropins or IVF - is presented.

## 2. Case Presentation

A 41½-year old woman presented with her husband (aged 41) for reproductive endocrinology consultation. They were both in good general health and took no regular medication. Her past surgical history was significant for an uncomplicated laparoscopic myomectomy in 2018. The assessment of endometrial cavity contour after the procedure was normal. At age 38, the patient was provisionally diagnosed with primary ovarian insufficiency (POI) based on repeatedly elevated levels of follicle-stimulating hormone (FSH) and “undetectable” anti-Müllerian hormone (AMH). The remainder of the work-up in this case was essentially unremarkable, including a normal endocrine profile and negative pregnancy test, although a mild thrombocytosis (platelets = 386K; normal range 150-379K) was discovered in the female. Moreover, two pregnancies were established without medical assistance > 5 yrs ago, but both were electively terminated without complication. The couple had initiated at least 10 IVF cycles elsewhere before consultation, however, none were successful; chart review attributed these failures to poor follicular response, “empty follicles,” fertilization failure, or culture arrest. Additional IVF attempts were discouraged, and for personal reasons the couple declined donor egg IVF.

**Figure 1:**
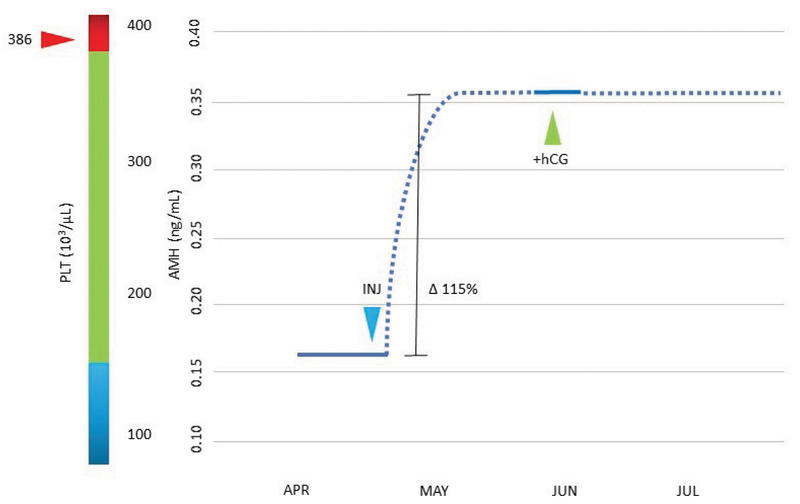
Serum AMH levels measured before versus after bilateral intraovarian injection of platelet-derived growth factors. Approximately six weeks after the treatment (blue arrow), an unassisted pregnancy was confirmed (green arrow). Baseline thrombocytosis is depicted at left (red arrow) to show nominally elevated pretreatment platelet concentration relative to the expected reference range (vertical bar). AMH: Anti-mullerian hormone; INJ: Intraovarian injection of enriched platelet growth factors; hCG: Human chorionic gonadotropin; PLT: Platelet concentration; Dashed/solid line: Estimated/verified data.

Here, the patient was counseled and a written informed consent was obtained for the injection of enriched autologous PDGFs into both ovaries. This treatment was offered as an extension of a previous IRB-approved prospective clinical trial (8). PDGFs were isolated first via obtaining autologous PRP activated by calcium gluconate as previously described (8), followed by additional enrichment by centrifugation at 250 g × 15 min with phosphate-buffered saline (Thermo Fisher Scientific, Carlsbad Calif USA) irrigation of the platelet pellet × 3. Thereafter, the resuspended platelet pellet was next processed through a centrifugation sequence (300 × g for 10 min, 2,000 × g for 10 min) to subtract debris, based on prior protocols (9-11). The resulting cell-free supernatant was maintained fresh at room temperature for ovarian insertion. After processing, a volume of approximately 1.5 mL was injected into ovarian stroma (subcapsular) under direct transvaginal ultrasound guidance with instrumentation as for conventional PRP dosing (5). The procedure was well tolerated with no complications, completed in < 10 min, and required no anesthesia or sedation. Using a uniform assay (12), the serum AMH was checked again the following month, and the level had increased from 0.163 ng/mL pretreatment to 0.352 ng/mL six weeks later (Figure 1). Ovulation occurred without medical assistance and serum human chorionic gonadotropin was subsequently noted at 804 mIU/mL. Her prenatal course was uneventful and she delivered a healthy 3,740 gr male infant without complication by elective cesarean at 39 weeks gestation. Both the mother and the baby continue to do well.

### Ethical consideration

Written informed consent was obtained from the patient, who read and approved the manuscript before publication.

## 3. Discussion

For both patients and providers, the problem of recurrent IVF failure is difficult and usually leads to discussion about donor oocytes. Although this approach is an established component of advanced fertility treatment since first introduced in the 1980s (13, 14), it is still sometimes unacceptable to some patients. For this reason, any advances to open safe and effective opportunities for patients would be welcomed. While successful pregnancy has been reported even when POI has been validated (15), the numerous IVF failures experienced by our patient focused renewed attention on intraovarian injection of autologous platelet-derived cytokines. Although no consensus exists regarding any preferred PRP sample preparation or injection technique, there is even less agreement regarding an optimal methodology for the newer approach for incubation and/or isolation of platelet growth factors.

**Figure 2:**
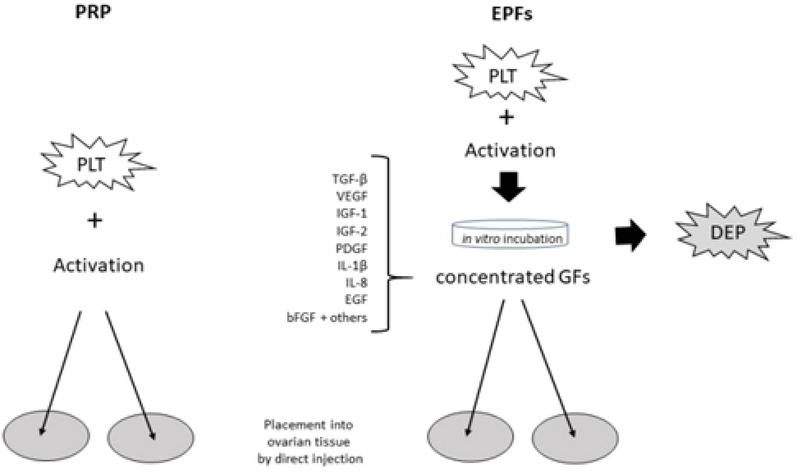
Outline comparing standard platelet-rich plasma (PRP) versus enriched platelet factors (EPF) ovarian treatment.

As summarized in Figure 2, selected platelet releasate components are shown with depleted platelets (DEP). These include TGF-β, a transcription activator modulating genes for differentiation, chemotaxis, and proliferation and activation of immune system cells; vascular endothelial growth factor, a signal protein stimulating blood vessel formation; insulin-like growth factors (1 and 2), proteins required for cell stimulation; PDGF, critical to blood vessel growth from adjacent capillaries, mitogenesis, and proliferation of mesenchymal cells including fibroblasts, osteoblasts, tenocytes, vascular SMCs, and mesenchymal stem cells; Interleukin-1β (IL-1β), an inflammatory marker involved in cell growth, differentiation, and programmed death; Interleukin-8 which initiates angiogenesis, perfusion, and movement to injury/infection sites; epidermal growth factor, a key messenger in cell proliferation, differentiation, and survival; as well as basic fibroblast growth factor, a mediator with mitogenic and cell survival activities including embryonic development, cell growth, morphogenesis, and tissue repair.

Placing such growth factors within ovarian tissue may potentiate higher AMH output and improve IVF response in several ways (16). One possibility is that any follicle emerging after intraovarian injection of these growth factors was merely latent, not completely absent. Perhaps more controversially, PDGFs could engage with uncommitted ovarian stem cells (17) and work along multiple signaling pathways to evoke differentiation to *de novo* oocytes. Indeed, PRP has been shown to induce proliferation of some cell populations to improve stemness and to enhance *in vitro* expression of receptivity markers (18, 19). Since these factors also have angiogenic properties, it is plausible that improving capillary flow and thus tissue oxygen delivery might induce beneficial ovarian effects after injection (20).

While it is tempting to ascribe any effects observed here to platelet cytokines, it is not possible to separate this component from the injection process itself where ovarian micro-puncture alone might be therapeutic (21, 22). However, we believe the link between the treatment and subsequent pregnancy here is supported by the brief interval between treatment and pregnancy, the absence of any other therapy, the serum AMH pattern after injection, and thrombocytosis. This latter issue draws notice to the role of platelet dynamics when ovarian PRP and related treatments are critically assessed; ambient platelet count has been identified as a modulator of AMH response independent of patient age, infertility duration, or pre-injection AMH level (5, 8).

We agree that limitations exist whenever case data are considered. For example, the marked uptick in serum AMH and the favorable reproductive outcome attained here after treatment are associative and not necessarily causative. Additional study should help clarify signaling pathways involved in follicular development, thereby providing potential techniques to make pregnancy possible even for older patients with low or absent ovarian reserve.

##  Conflict of Interest

ESS and SHW have received a provisional U.S. patent for the process & treatment of ovarian disorders using platelet cytokine derivatives. NSR has no conflicts to disclose.
